# Data set showing the development of a hyperspectral imaging technique using LA-ICP-MS to determine the spatial distribution of nutrients in soil cores

**DOI:** 10.1016/j.dib.2021.107677

**Published:** 2021-12-05

**Authors:** Muhammad Zaeem, Muhammad Nadeem, Thu Huong Pham, Waqar Ashiq, Waqas Ali, Syed Shah Mohioudin Gillani, Eric R.D. Moise, Heather Leier, Vanessa Kavanagh, Lakshman Galagedara, Mumtaz Cheema, Raymond Thomas

**Affiliations:** a School of Science and the Environment, Grenfell Campus, Memorial University of Newfoundland, Corner Brook, A2H 5G4, Canada; b Natural Resources Canada, Canadian Forest Service - Atlantic Forestry Centre, 26 University Drive, Corner Brook, Newfoundland and Labrador, Canada, A2H 5G4; c Department of Art, University of Calgary Alberta, Canada, T2N 1N4; d Department of Fisheries, and Land Resources, Government of Newfoundland and Labrador, A0L 1K0, Canada; e School of Environmental Sciences, University of Guelph, N1G 2W1, Guelph, Ontario, Canada

**Keywords:** Elemental imaging, Soil core nutrient imaging, Nutrient mapping, Spatial mineral distribution, Imaging mass spectrometry

## Abstract

This data in brief article represents the data set associated with a research article published in Geoderma [1]. The data set represents figures showing the spatial distribution of selected macro and micronutrients, and their quantification in different crop or nutrient management systems practiced in the boreal ecosystem. Spatial distribution of nutrients was measured by laser ablation inductively coupled plasma mass spectrometry (LA‒ICP‒MS), using the new techniques we developed to visualize nutrient distribution in intact soil cores representative of the root rhizosphere. This data article supports the findings published in the main article [1]. This work also demonstrates that LA-ICP-MS is a valuable technique to image the spatial distribution of macro and micronutrients in intact soil cores as affected by different crop management practices.

**Specifications Table**

**Table:** 

Subject	Agriculture, Environmental Science, Natural Resource Management
Specific subject area	Hyperspectral imaging of nutrients in soil cores using LA-ICP-MS and complimentary quantification with ICP-MS
Type of data	Figures
How data was acquired	Soil core samples were collected with a soil auger and core sampler (AMS, Inc. USA); plastic liners (Osprey Scientific Inc. AB, Canada). Soil cores ablated using a laser ablation system. Instruments: LA-ICP-MS (ESI NWR 213, Nd-YAG, Elemental Scientific Lasers, MT, USA) and ICP-MS (iCAP Q. Thermo Scientific, ICP-MS, ON, Canada). Cores were spiked with internal standards consisting of Rhodium and Indium (0.1 mg *L* ^−1^). Image processing of the cores completed using: Iolite Version 3.4 Statistical analysis was conducted using the Statistix-10 software program (Analytical Software, FL, USA); SigmaPlot (Systat Software Inc., San Jose, CA).
Data format	Data is in raw and analyzed form. An Excel file with data set has been uploaded with the article.
Parameters for data collection	Operating conditions for laser ablation (ESI NWR 213, Nd-YAG) included laser wavelength 213 nm with 220 µ ms^−1^ scan speed, 7 mJ laser energy applied, helium gas flow 800 mL min^−1^, circular spot size 100 µm, and 20 repetitions in continuous mode with helium as a carrier gas. ICP-MS (iCAP Q. Thermo Scientific, ICP-MS) operating conditions included auxiliary gas flow @ 0.79 L min^−1^, nebulizer gas flow @ 1.01 L min^−1^, plasma gas flow @ 14 L min^−1^, RF power 1548 W, STD detector mode, and 0.01 s dwell time with auto lens calibrated.
Description of data collection	Intact cores were spiked with internal standards and ablated using the parameters indicated above. The acquired data was obtained from the ICP MS software (iCAP Q. Thermo Scientific, ICP-MS). The Iolite software was used to generate the images for each respective element selected during the ablation process (See experimental design section below for detailed step by step procedures for creating the images). This approach produced high resolution images showing the spatial distribution of select nutrients in the intact soil cores. The second half of the core was used to quantify the nutrients present in the core. The soil was digested in concentrated nitric acid using a microwave digestion system using the following parameters: ramped temperature @ 180 °C for 10 min with 100% power and held at this temperature for 20 min to allow complete sample digestion. The concentration of the elements in the core was determined using a 43 element standard mix (IV‒ICPMS‒71A) obtained from Inorganic™ Ventures, Inc. (Christiansburg, VA 24,073, USA) prepared at the following concentration: (10, 20, 50, 100, 200, 300, and 500 ppb). This information was used to relate the quantity of the nutrients determined in the intact soil core
Data source location	Research Site: Pynn's Brook Agricultural Research Station City/Town/Region: Pasadena, Newfoundland Country: Canada Latitude and longitude: 49.0130° N, 57.5894° W Institution: Grenfell Campus, Memorial University of Newfoundland City/Town/Region: Corner Brook, Newfoundland Country: Canada Latitude and longitude: 49° 4′21.93″N 57°33′36.51″W
Data accessibility	Data is available within this article and the raw data files in excel format also uploaded on public repository and doi (https://data.mendeley.com/datasets/fvhwgwvfxc/1) is provided as the supplementary data.
Related Research Articles	M. Zaeem, M. Nadeem, T. Huong Pham, W. Ashiq, W. Ali, S. Shah Mohioudin Gillani, E.R.D. Moise, H. Leier, V. Kavanagh, L. Galagedara, M. Cheema, R. Thomas, Development of a hyperspectral imaging technique using LA-ICP-MS to show the spatial distribution of elements in soil cores, Geoderma. 385 (2021) 114,831. https://doi.org/10.1016/j.geoderma.2020.114831.

## Value of the Data

• This data set is useful in facilitating fellow scientists in the community to either replicate or improve the novel method to discern the spatial distribution of multiple elements in intact soil cores.
• This data provides baseline information as well as analytical output that can be used to guide future experiments.
• We think this data will be useful to facilitate repetition of this study as well as to enable new studies to improve upon the method presented.
• Multidisciplinary data set obtained to evaluate different agronomic practices used during forage production in a boreal agro-ecosystem. The approach is applicable to any land management system.
• Data set will enhance our understanding of the spatial distribution of nutrients or nutrient cycling in the rhizosphere as affected by different crop management systems.
• Data set could also help to examine contaminated soils and help guide decisions in choosing the best remedies to alleviate the harmful elements from contaminated soils and the environment.
• Additionally, the developed technique could be very useful in assessing the effects of crop management systems and fertilizer application on the spatial distribution of nutrients in the plant root zone, as well as inform or guide the best decisions for land use and management strategies.

## 1 Data Description

**Fig. 1:**
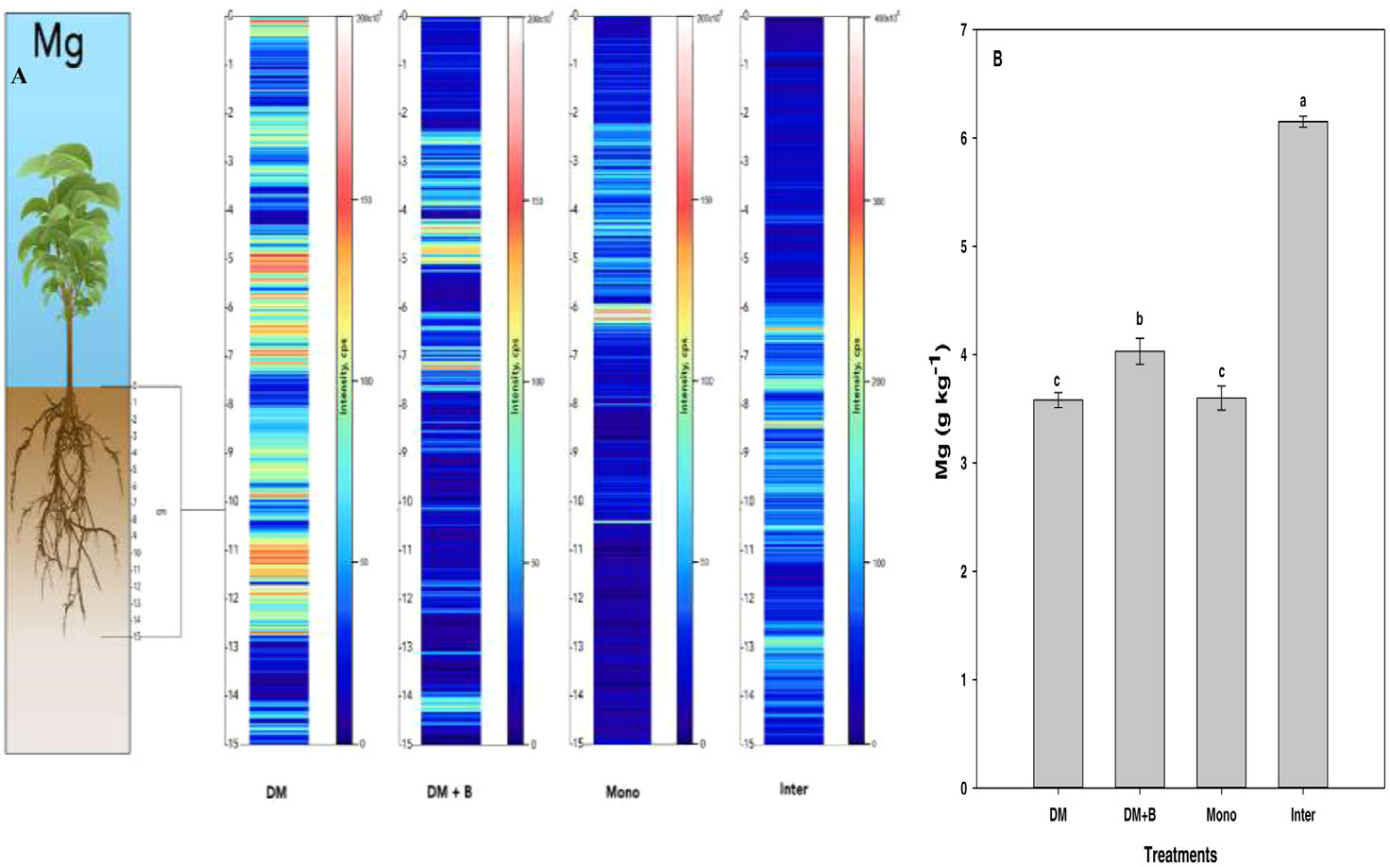
Qualitative images (A) showing the spatial distribution of magnesium (Mg), and its bulk quantification averaged over the entire sample length (B) in different nutrient or crop management systems as measured by LA-ICP-MS and ICP-MS, respectively. Values (g kg^−1^) represent means ± standard error for four replications. Different letters in the bar chart denote statistically significant differences (*p* < 0.05) in soil Mg concentration based on mean comparison using Fisher's LSD test. DM = dairy manure, DM+*B* = dairy manure amended with biochar, Mono = monocropping, Inter = intercropping.

**Fig. 2:**
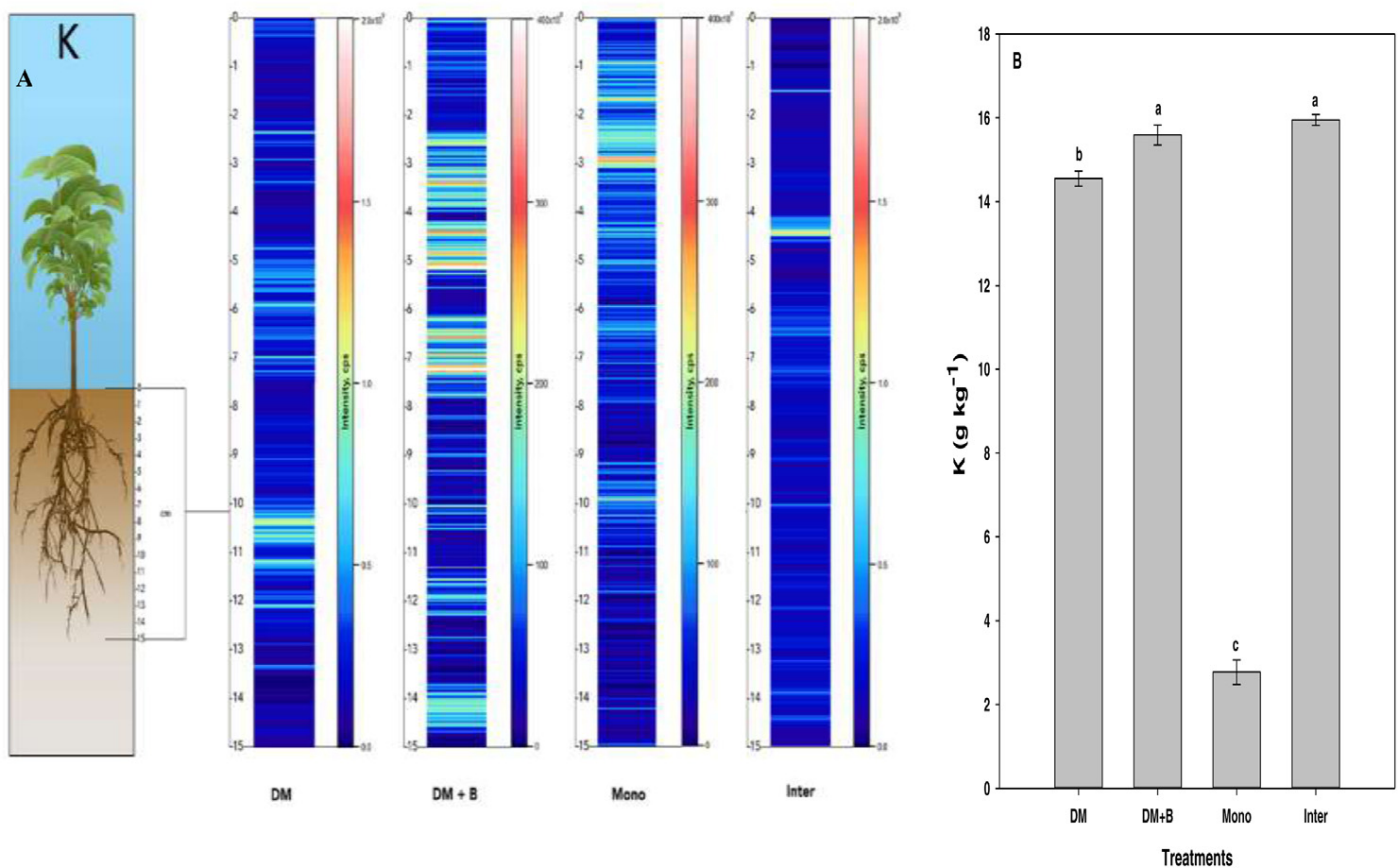
Qualitative images (A) showing the spatial distribution of K, and its bulk quantification averaged over the entire sample length (B) in different nutrient or crop management systems as measured by LA‒ICP‒MS and ICP‒MS, respectively. Values (g kg^−1^) represent means ± standard errors (*n* = 4). Different letters denote statistically significant differences (*P*<0.05) in soil potassium concentration based on mean comparisons using Fisher's LSD test. DM = dairy manure, DM+*B* = dairy manure amended with biochar, Mono = monocropping, Inter = intercropping.

**Fig. 3:**
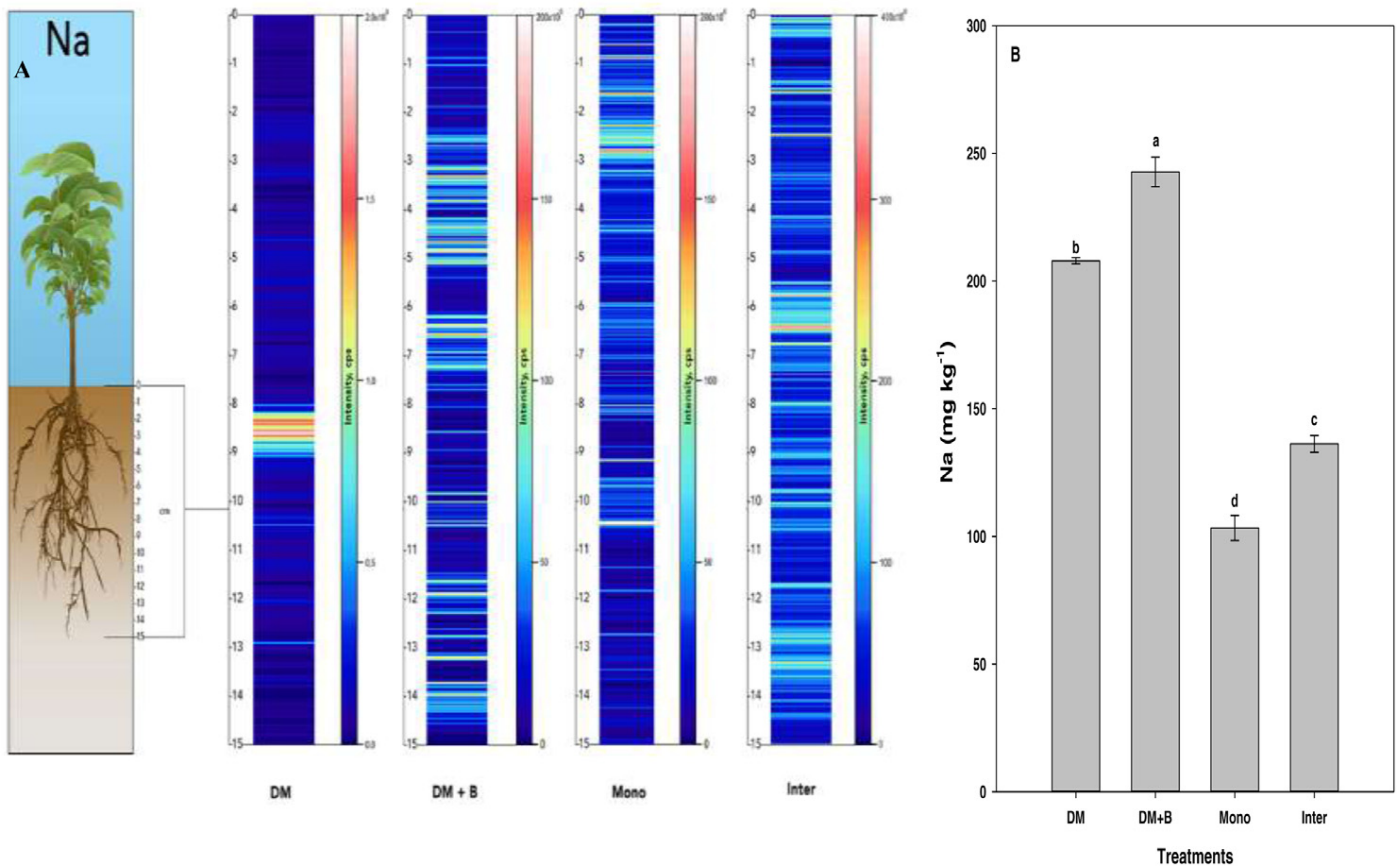
Qualitative images (A) showing the spatial distribution of Na, and its bulk quantification averaged over the entire sample length (B) in different nutrient or crop management systems as measured by LA‒ICP‒MS and ICP‒MS, respectively. Values (mg kg^−1^) represent means ± standard errors (*n* = 4). Different letters denote statistically significant differences (*P*<0.05) in soil sodium concentration based on mean comparisons using Fisher's LSD test. DM dairy manure, DM+*B* = dairy manure amended with biochar, Mono = monocropping, Inter = intercropping.

**Fig. 4:**
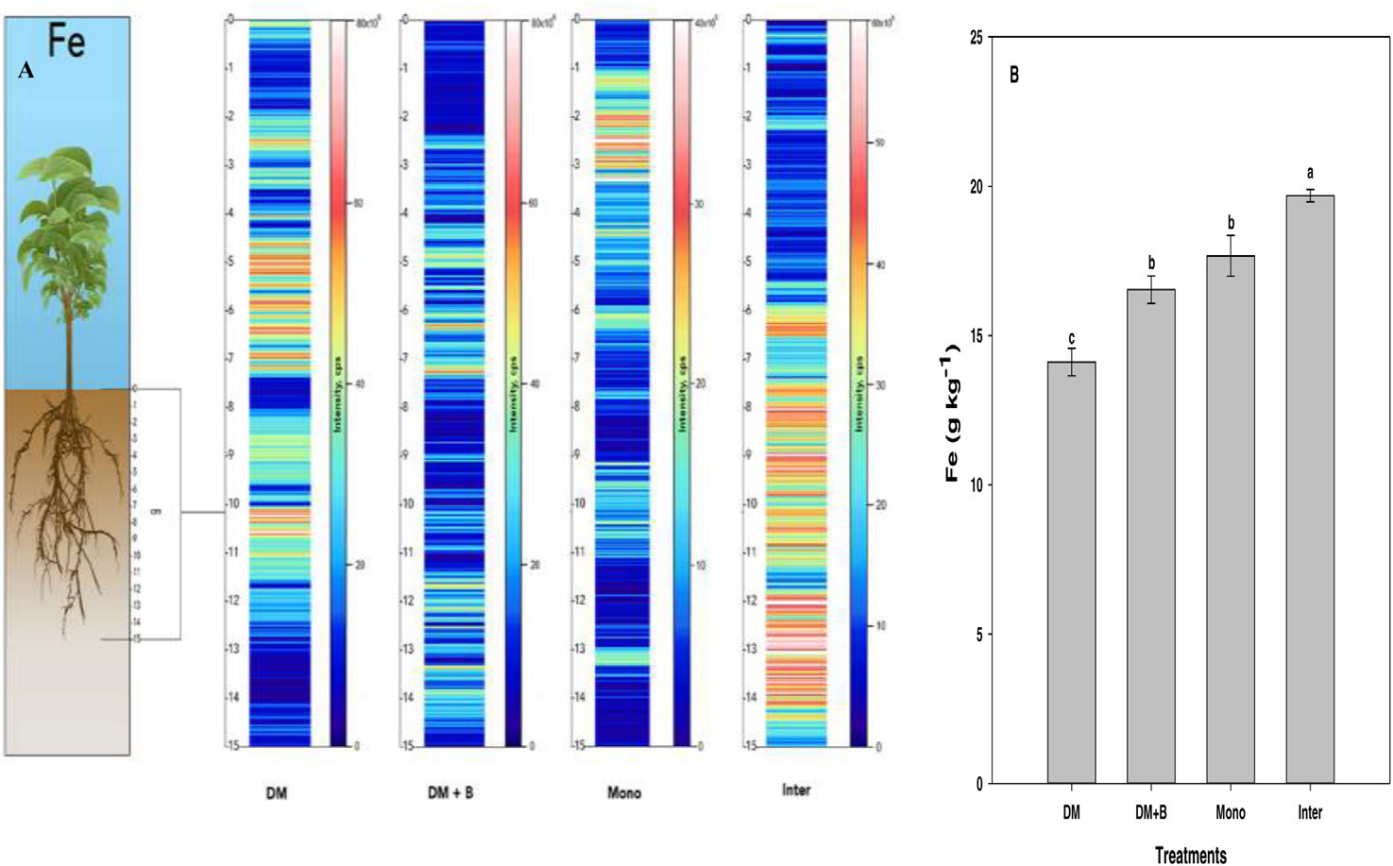
Qualitative images (A) showing the spatial distribution of Fe, and its bulk quantification averaged over the entire sample length (B) in different nutrient or crop management systems as measured by LA‒ICP‒MS and ICP‒MS, respectively. Values (mg kg^−1^) represent means ± standard errors (*n* = 4). Different letters denote statistically significant differences (*P*<0.05) in soil iron concentration based on mean comparisons using Fisher's LSD test. DM = dairy manure, DM+*B* = dairy manure amended with biochar, Mono = monocropping, Inter = intercropping.

**Fig. 5:**
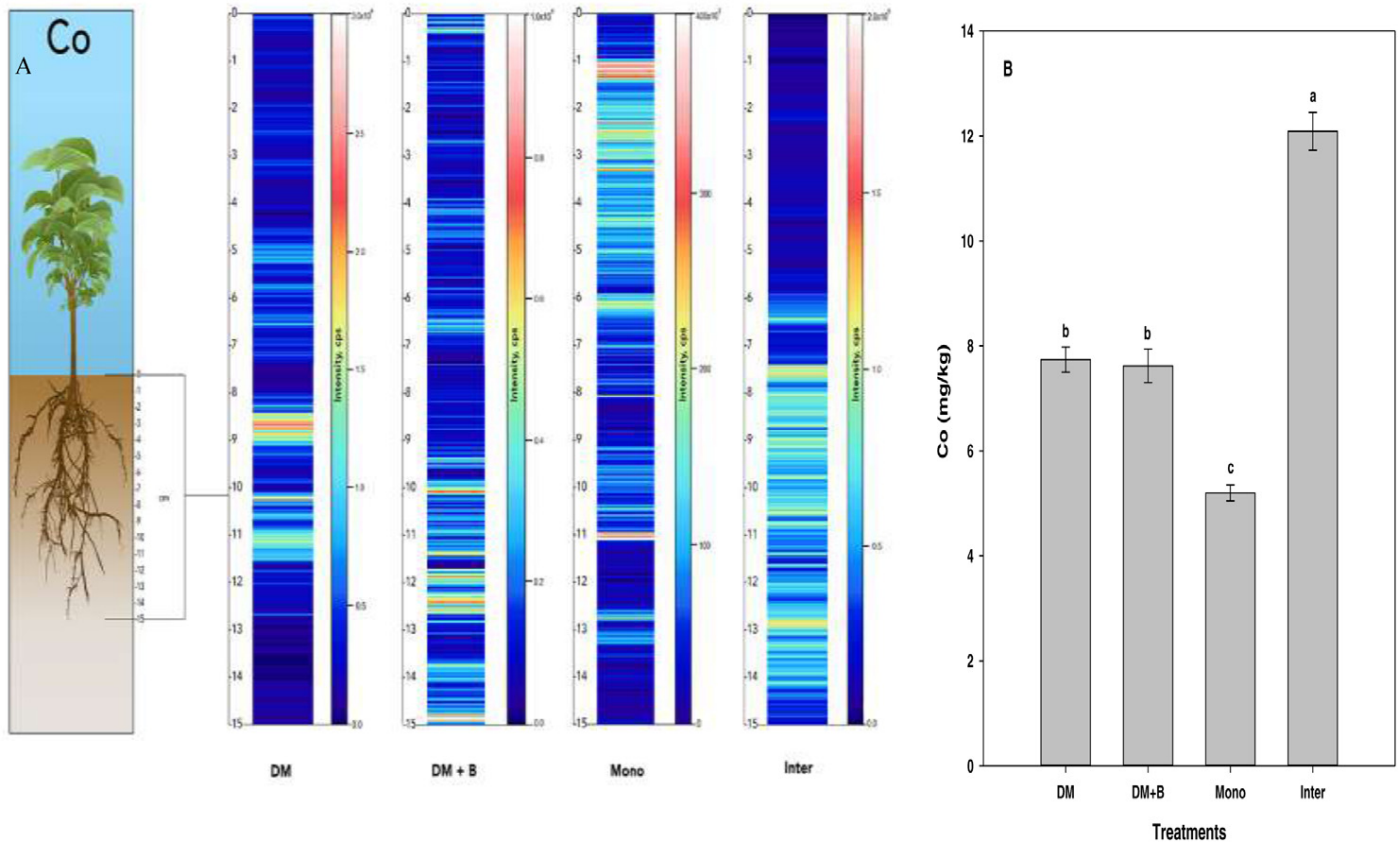
Qualitative images (A) showing the spatial distribution of cobalt (Co), and its bulk quantification averaged over the entire sample length (B) in different nutrient or crop management systems as measured by LA‒ICP‒MS and ICP‒MS, respectively. Values (mg kg^−1^) represent means ± standard errors (*n* = 4). Different letters denote statistically significant differences (*P*<0.05) in soil cobalt concentration based on mean comparisons using Fisher's LSD test. DM = dairy manure, DM+*B* = dairy manure amended with biochar, Mono = monocropping, Inter = intercropping.

Laser ablation inductively coupled plasma mass spectrometry (LA-ICP-MS) is a very efficient and sensitive surface nutrient imaging technique that can be used to discern the spatial distribution of metals and non-metals in biological and environmental samples [1]. This data set contains information regarding the effect of four different agronomic practices on the spatial distribution of nutrients in the plant root zone. Nutrients were extracted using acid digestion and quantified using ICP-MS. A multi-element (43 elements) high purity ICP-MS standard solution was used for external calibration as described above in the specification table during quantitative analysis. A seven-point calibration was done for each of the different elements with a range of 0- 100 ppb and R^2^ values ranging from 0.988 to 0.998 for each element. The data set contains five figures demonstrating the imaging and spatial distribution of mineral nutrients in intact soil cores using LA-ICP-MS. Figs. 1–5 demonstrate the effects of different crop management practices on the spatial distribution of magnesium, potassium, sodium, iron, and cobalt in the plant rhizosphere.

## 2 Experimental Design, Materials and Methods

A field experiment was conducted at Pynn's Brook Agricultural Research Station, Pasadena, Newfoundland (NL) (49.0130° N, 57.5894°W), Canada. Forage soybean and silage corn crops were planted with the SAMCO system (SAMCO 2200 Agricultural Manufacturing, Limerick, Ireland) under field conditions in a randomized complete block design. Crops were either sown alone (monocropping) or intercropped with different nutrient management practices including i) dairy manure (DM) and ii) dairy manure + biochar (DM+*B*). DM was applied @ 30,000 L ha^−1^ according to local dairy farmer practice and was thoroughly mixed before crop seeding. To assess the spatial distribution of minerals affected by crop or nutrient management practices, intact soil core samples were collected at crop maturity and frozen at −20 °C. Intact soil core samples were collected using 3.8 cm × 15.0 cm plastic liners (Osprey Scientific Inc. Edmonton, AB. Canada). Hyperspectral images of the frozen samples (3.8 cm × 7.5 cm dimension) were taken using LA-ICP-MS, whereas acid digested samples were analyzed with ICP-MS for quantitative analysis. For imaging purpose, each core sample was cut into two equal halves (7.5 cm each) and then split longitudinally to fit in the LA drawer. The dimensions of each core were 3.75 cm *L* × 7.5 cm W. Qtegra Intelligent Scientific Data Solution Software was used to operate and collect LA-ICP-MS data, as well as qualitative and quantitative elemental data output. Iolite Software was used to process the LA-ICP-MS data output into high-resolution spatial images. The raw data files in excel format are uploaded on public repository and doi (https://data.mendeley.com/datasets/fvhwgwvfxc/1) is provided as the supplementary data. The created data files obtained from Qtegra software were imported to the Iolite software (Iolite Version 3.4) which is an add-on to Igor Pro (WaveMetrics, Inc. Igor Pro 6.37) as a universal file type (CSV) format. The steps involved in image construction are given below:

Raw signal intensity data were exported from Qtegra as a .csv file. The file was directly imported in Iolite and processed (converted) into an image map via the following steps: i) matching to the corresponding laser log file; ii) selection of sample and baseline regions; iii) gas blank subtraction; iv) data reduction based on [mention thresholds]; v) cell space image creation; vi) selection of the element (channel) for visualization; vi) Selected a color palette vii) export of the image as in PNG format.

## CRediT Author Statement

**Muhammad Zaeem:** Conducted the field experiments and wrote the first draft of the manuscript; **Muhammad Nadeem** and **Thu Huong Pham:** Helped in study planning and experimental design; **Waqar Ashiq, Waqas Ali** and **Syed Shah Mohioudin Gillani:** Helped in data acquisitions and lab analyses; **Eric R.D. Moise** and **Heather Leier:** Helped in the data visualization of analysis; **Mumtaz Cheema** and **Lakshman Galagedara:** Assisted in study conceptualization and co-supervised the project; **Vanessa Kavanagh** Helped in field experimentation and revising the manuscript; **Raymond Thomas** Conceptualized the project and is the principal investigator (PI). All authors contributed to manuscript revision, read, and approved the revised version.

## Declaration of Competing Interest

The authors declare that they have no known competing financial interests or personal relationships which have or could be perceived to have influenced the work reported in this article.
